# What Are the Most Effective Behavioural Strategies in Changing Postpartum Women’s Physical Activity and Healthy Eating Behaviours? A Systematic Review and Meta-Analysis

**DOI:** 10.3390/jcm9010237

**Published:** 2020-01-16

**Authors:** Siew Lim, Briony Hill, Stephanie Pirotta, Sharleen O’Reilly, Lisa Moran

**Affiliations:** 1 Monash Centre for Health Research and Implementation, Monash University, Clayton, VIC 3168, Australia; briony.hill@monash.edu (B.H.); stephanie.pirotta@monash.edu (S.P.); lisa.moran@monash.edu (L.M.); 2 School of Agriculture and Food Science, University College Dublin, Belfield, Dublin, Ireland; sharleen.oreilly@ucd.ie

**Keywords:** postpartum women, lifestyle, weight management, systematic review, behaviour strategies

## Abstract

Successful implementation of postpartum lifestyle interventions first requires the identification of effective core components, such as strategies for behavioural change. This systematic review and meta-analysis aimed to describe the associations between behavioural strategies and changes in weight, diet, and physical activity in postpartum women. Databases MEDLINE, CINAHL, EMBASE, and PsycINFO were searched for randomised controlled trials of lifestyle interventions in postpartum women (within 2 years post-delivery). Strategies were categorised according to the Behaviour Change Technique Taxonomy (v1). Forty-six articles were included (n = 3905 women, age 23–36 years). Meta-analysis showed that postpartum lifestyle interventions significantly improved weight (mean difference −2.46 kg, 95%CI −3.65 to −1.27) and physical activity (standardised mean difference 0.61, 95%CI 0.20 to 1.02) but not in energy intake. No individual strategy was significantly associated with weight or physical activity outcomes. On meta-regression, strategies such as problem solving (β = −1.74, P = 0.045), goal setting of outcome (β = −1.91, P = 0.046), reviewing outcome goal (β = −3.94, P = 0.007), feedback on behaviour (β = −2.81, P = 0.002), self-monitoring of behaviour (β = −3.20, P = 0.003), behavioural substitution (β = −3.20, P = 0.003), and credible source (β = −1.72, P = 0.033) were associated with greater reduction in energy intake. Behavioural strategies relating to self-regulation are associated with greater reduction in energy intake.

## 1. Introduction

Weight retention or weight gain after birth is a key contributor to obesity in women of reproductive age. On average, women retain 0.5 to 3.5 kg from each pregnancy, although up to half of all women have significant weight retention of 4.5 kg or more above their preconception weight by 1 year after birth [1,2,3,4]. This results in about one-third of all women who had a healthy body mass index (BMI) prior to pregnancy developing overweight or obesity by one year after birth [3]. Retaining or gaining excess weight after birth is associated with significant short- and long-term maternal and child health risks. Women who gained 3 or more BMI units within 2 years between pregnancies were at 60% to 80% increased risk of stillbirth and large-for-gestational-age babies in subsequent pregnancies [5]. Failure to return to prepregnancy weight by 6 months postpartum also increases the risk of significant weight gain in the following decade [6,7]. Weight gain after birth also increases the risk of developing chronic diseases, including type 2 diabetes in women with previous gestational diabetes [8]. The time after birth is, therefore, a critical window to intervene for maternal weight management.

The implementation of complex interventions, such as lifestyle (dietary and physical activity) behavior change interventions for postpartum women, into various settings requires the identification of the core components that are associated with intervention effectiveness [9]. These core components form the “active ingredients” of the intervention, while the other intervention characteristics form the adaptable periphery to suit the contextual needs of a given setting [9]. We have previously identified some elements of the core components for postpartum lifestyle interventions using the Template for Intervention Description and Replication (TIDieR) framework, which identifies intervention characteristics such as the what, when, where, who, how, and how much (i.e., dose) associated with the greatest effects [10]. We found that the combination of diet and physical activity advice along with delivery by health professionals were associated with significantly greater weight loss in postpartum women [10]. However, significant heterogeneity remained in the subgroups, suggesting other factors could be associated with the effectiveness of these interventions. Behavioural strategies within the interventions are potentially key contributing factors to intervention effectiveness [11,12,13,14].

A number of studies have previously investigated the associations between behavioural strategies and intervention effects through meta-analyses and meta-regressions in the general population [11,12,13,14]. Strategies such as the provision of instructions, self-monitoring, relapse prevention, and prompting practice were associated with better outcomes in improving dietary and/or physical activity behaviours in adults with obesity [12]. Previous meta-regressions have also considered the group effect of strategies that are congruent with the Control Theory such as self-monitoring, monitoring, and feedback and found them to be associated with greater effect sizes in weight and lifestyle interventions [12,13]. While some behavioural strategies may be effective for all population groups (e.g., self-monitoring) [13], previous studies suggest that specific strategies may apply to certain subgroups, such as home environment restructuring to support changes in weight-related diet and physical activity behaviours for children [15] and social support for older adults to increase fruit and vegetable intakes [16]. Postpartum women face unique health behaviour change challenges such as ongoing sleep deprivation or tiredness, financial constraints due to changed work circumstances, lack of time due to infant care, low motivation for self-care, lack of support from family and friends, changes in priorities, and the demands of childcare [17,18,19]. It is unclear if unique behavioural strategies may be associated with greater effects in diet and physical activity interventions for postpartum women.

This study describes a systematic review and meta-analyses of behavioural strategies used in lifestyle interventions for postpartum women (up to 2 years after birth). The study’s primary aim was to describe the associations between behavioural strategies and weight loss in postpartum women. The secondary aim was to describe the associations between behavioural strategies and key lifestyle factors associated with weight loss (i.e., diet (energy intake) and physical activity) in postpartum women. 

## 2. Methods

The systematic review and meta-analysis adhered to the Preferred Reporting Items for Systematic Reviews and Meta-Analyses (PRISMA) Statement [20] (Table S1). It was registered at PROSPERO (CRD42018086206), and the review protocol is available at http://www.crd.york.ac.uk/PROSPERO/display_record.php?ID=CRD42018086206.

Thirteen databases were systematically searched on 3 May 2019: MEDLINE, EMBASE, Pubmed, PsycINFO, CINAHL, Cochrane Database of Systematic Reviews, American College of Physicians Journal Club, Database of Abstracts of Reviews of Effects, Cochrane Central Register of Controlled Trials, Cochrane Pregnancy and Childbirth Group Trials Register, Cochrane Methodology Register, Health Technology Assessment, and National Health Service Economic Evaluation Database. Search terms are shown in Table S2. Studies were not limited by date or language and translations were sought where possible.

### 2.1. Study Selection

The inclusion and exclusion criteria are as shown in Table S3. Randomised controlled trials (RCTs) were eligible for inclusion if they involved lifestyle modification (diet, physical activity, or behavioural therapy) for postpartum women (within 2 years of delivery) [21] with minimal intervention control groups (no more than single session at baseline). Studies were excluded if they did not involve lifestyle modification that could result in weight loss, for example, allergen avoidance studies, acute studies (e.g., post-physical activity breast milk composition), supplement trials (e.g., fish oil), or breastfeeding interventions that focused only on infant feeding. Studies were also excluded if recruitment or implementation commenced during pregnancy, except if a separate intervention group commencing in the postpartum period could be independently assessed. Studies that did not report any relevant outcomes in terms of body weight, body mass index (BMI), energy intake, or physical activity were excluded. The following publication types were excluded: editorials, reviews, conference abstracts, letters, commentaries, uncontrolled trials, non-randomized trials, and study protocols. Abstracts, titles, and full texts were independently screened by two out of three reviewers (C.C. and S.L./S.O.R.). Consensus and arbitration occurred where needed to arrive at the final decisions of the screening process (S.L., C.C., S.O.R., and B.H.).

### 2.2. Data Extraction

Two reviewers independently extracted the following data using a data extraction template: general characteristics of the study (author, year of publication, study country, sample size, and intervention type), participants (BMI at inclusion, time since birth at baseline, and parity), and outcomes (body weight, BMI, energy intake, and physical activity). Behavioural strategies were identified and categorized using the Behaviour Change Technique Taxonomy version 1 (BCTTv1) [22]. Only techniques targeted at changing the diet, physical activity, or weight of postpartum women were considered. The 93 strategies listed in BCTTv1 were rated as present or absent. Both the intervention and control groups were coded. Only behavioural strategies that were present in the intervention group and absent in the control group were included in the analyses. This approach is consistent with past systematic reviews and meta-regressions of behavioural strategies [11]. Coders completed the BCTTv1 coding online training course developed by the BCTTv1 authors. The coders were dietitians experienced in lifestyle intervention development for women of reproductive age (S.L. and L.M.). One reviewer (S.L.) independently coded all studies while a second reviewer (L.M.) independently coded 10% of randomly selected studies. Inter-reviewer agreement of 96% (Cohen’s kappa = 0.76) was achieved, and any discrepancies were resolved by consensus and arbitration (S.L., L.M., and B.H.).

### 2.3. Risk of Bias Assessment

The Revised Cochrane Risk of Bias Tool for Randomized Trials (RoB 2.0) was used to assess the risk of bias in each included study [23]. This tool evaluated bias from the domains of randomisation, deviations from the intended protocol, missing outcome data, measurement of the outcome, and selective reporting. Overall ratings of low, some concerns, or high risk of bias were calculated for each study. One reviewer (X.L.) independently appraised all the studies while a second reviewer (C.C.) independently appraised 10% of randomly selected studies. Inter-reviewer agreement of 83% was achieved (Cohen’s kappa = 0.67). Discrepancies were resolved by consensus and arbitration (X.L., C.C., and S.L.).

### 2.4. Data Synthesis and Analysis

Outcomes were expressed as mean differences (MDs) or standardised mean differences (SMDs) with 95% confidence intervals (CIs). Outcomes were combined in the meta-analysis with the inverse variance random-effects model (restricted maximum-likelihood estimator for tau-squared) due to clinical or statistical heterogeneity [24]. Chi-square test was used to assess heterogeneity between the studies (P < 0.1 was considered statistically significant). Inconsistency between the studies was described by the *I*^2^ tests. Sources of heterogeneity by the total number of behavioral strategies, individual behavioral strategy, and behavioral strategies congruent to Control Theory (i.e., all the behavioral strategies on goals and planning and feedback and monitoring, Table S4) were explored in univariate meta-regression for weight, diet, and physical activity outcomes to allow comparison with previous meta-analyses of behavioral strategies [12]. Funnel plots and Egger’s tests were conducted to determine publication bias. Data analysis was performed using RStudio 1.1.463 (Free Software Foundation, Inc. 1991,1999, Boston, USA).

## 3. Results

### 3.1. Identification of Studies

**Figure 1:**
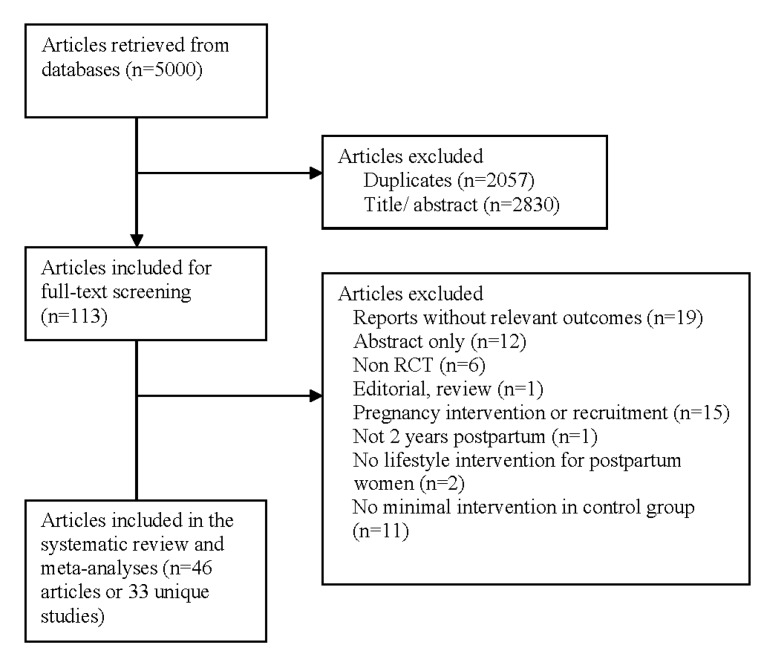
Flowchart of studies included.

The search identified 5000 articles, as shown in Figure 1. After an initial title and abstract screening, 113 full-texts were considered further, of which 46 articles representing 33 unique studies (*n* = 3905 women, age 23–36 years) met the review’s inclusion criteria. The reasons for exclusion are as shown in Figure 1.

### 3.2. Study Characteristics

The included studies characteristics are shown in Table 1. Most studies were conducted in developed countries: USA (*n* = 14), Australia (*n* = 4), UK (*n* = 3), Canada (*n* = 2), Greece (*n* = 1), Sweden (*n* = 2), and Japan (*n* = 1). The remaining were conducted in developing countries such as Iran (*n* = 3), Israel (*n* = 1), Taiwan (*n* = 1), and Thailand (*n* = 1). One study involved a diet-only intervention, twenty studies included combined diet and exercise interventions, while twelve studies had exercise-only interventions (Table 1). Sample size ranged from 24 [25] to 542 [26,27]. Study duration ranged from 11 days [28] to 15 months [26].

### 3.3. Participants

The risk factors of participants at inclusion are as shown in Table 1. Sixteen studies included only women with overweight or obesity defined as having excess BMI at baseline, excess BMI prepregnancy, or excess postpartum weight retention (Table 1). Time after birth at baseline ranged from 3 weeks [29] to 18 months [26]. Three studies included only primiparous women [30,31,32]. The average age of participants ranged from 23 [31] to 36 years [33]. Twenty-two studies reported ethnicity of the participants, with 13 studies included mostly Caucasian participants (Table S5).

### 3.4. Risk of Bias Assessment

The overall risk of bias assessment for all included studies is shown in Table S6. All studies had a high overall risk of bias except for one [34]. The overall high risk of bias was primarily due to deviations from intended interventions. Ten studies reported intention-to-treat analyses [26,34,35,36,37,38,39,40,41,42,43], which reduces the risk of bias from missing outcome data. Two studies [44,45,46,47] were evaluated to have a high risk of bias for missing outcome data due to a higher dropout rate in the intervention group, which could be related to the true outcome. One-third of the studies was assessed as having a low risk of bias on “measurement of outcome” due to the use of objective measures such as step counts from pedometers. Two studies were at risk of selective reporting due to redundant methods of analyses of a single outcome [43,48]. Funnel plots of body weight and energy intake were largely symmetrical, and Egger’s tests suggested low risk of publication bias (Figure S1). Funnel plot of physical activity suggested publication bias, with small studies finding a decrease in physical activity not being published (Egger’s test P = 0.008) (Figure S1).

### 3.5. Meta-Analysis of the Effect of Lifestyle Intervention on Postpartum Women

Lifestyle interventions significantly improved body weight (MD −2.46 kg, 95% confidence interval, CI −3.65 to −1.27, 25 studies, 1945 participants, *I*^2^ = 79%) and physical activity (SMD 0.61, 95% CI 0.20 to 1.02, 24 studies, 2138 participants, *I*^2^ = 86%) compared with control groups. Energy intake was not significantly different (MD −605 KJ/day, 95% CI −1530 to 320, 12 studies, 1123 participants, *I*^2^ = 82%). Significant statistical heterogeneity was present for all outcomes. The forest plots of these meta-analyses are as shown in Figure S1.

### 3.6. Meta-Regression of Behavioural Strategies

**Figure 2:**
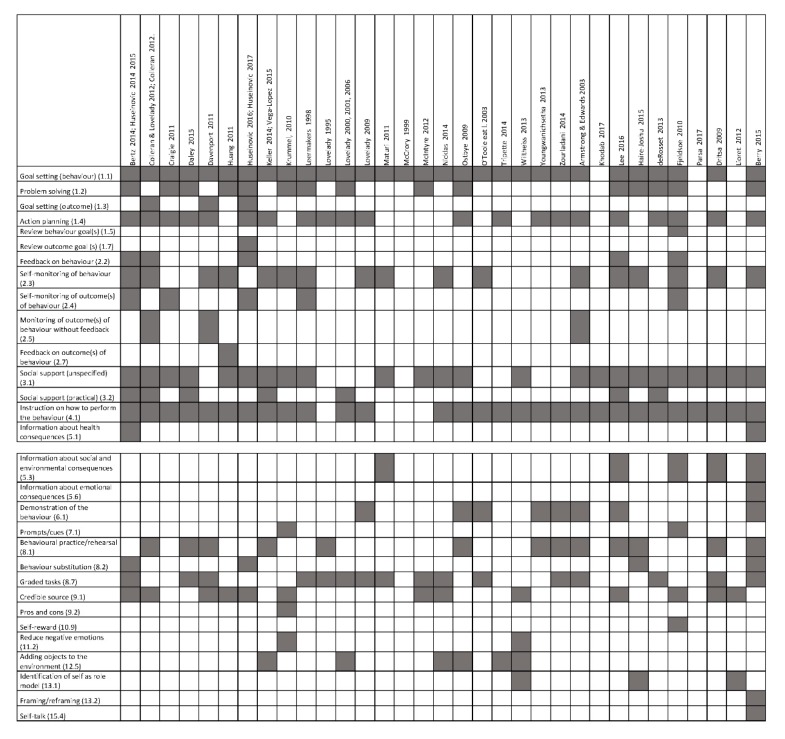
Behavioural strategies in lifestyle interventions in postpartum women.

The behavioural strategies within each included study are shown in Figure 2. Thirty-three different behavioural strategies were coded for the included studies of lifestyle intervention in postpartum women. The average number of behavioural strategies used in an intervention was 7.0 ± 3.0 (range: 1 to 15).

#### 3.6.1. Effect of Total Number of Behavioural Strategies

Inclusion of a greater number of behavioural strategies in an intervention was associated with greater decrease in energy intake (Table 2). Total number of behavioural strategies was not significantly associated with weight (Table S7) or physical activity (Table S8).

#### 3.6.2. Effect of Individual Behavioural Strategies

The behavioural change strategies of problem solving, goal setting of outcome, reviewing outcome goal, feedback on behaviour, self-monitoring of behaviour, behavioural substitution, and credible source were associated with greater decreases in energy intake (Table 2). No individual strategies were significantly associated with weight (Table S7) or physical activity (Table S8).

#### 3.6.3. Effect of Behavioural Strategies Congruent to Control Theory

Studies with behavioural strategies congruent to Control Theory had greater decreases in energy intake (Table 2). Provision of behavioural strategies congruent to Control Theory was not significantly associated with weight (Table S7) or physical activity (Table S8).

## 4. Discussion

### 4.1. Principal Findings

As previously reported, we found that lifestyle interventions in postpartum women resulted in significant improvements in weight and physical activity [48]. We reported that the total number of behavioural strategies and the provision of certain behavioural strategies including problem-solving, goal-setting of outcome, reviewing outcome goal, feedback on behaviour, self-monitoring of behaviour, behavioural substitution, and credible source were associated with greater decreases in energy intake. Studies with behavioural strategies congruent with Control Theory also had greater decreases in energy intake. However, none of the behavioural strategies explained the heterogeneity in weight or physical activity outcomes.

### 4.2. Interpretation of Findings

This review aimed to identify active ingredients in lifestyle interventions for postpartum women by analysing behavioural strategies that were significantly associated with lifestyle or weight-related outcomes, such as weight, energy intake, and physical activity. A similar analysis was previously conducted to identify the active implementation ingredients for diabetes care [49]. The identification of intervention core components is an important evidence synthesis step in the implementation of primary research [9]. This enables necessary adaptations to occur when developing interventions across different settings without omitting any outcome active ingredients. For example, we previously adapted an intervention for postpartum women by altering the delivery format while preserving the core components, which enabled the replication of the study in a different setting without a loss of effect size [50]. To our knowledge, this is the first review to identify core behavioural strategies in postpartum lifestyle interventions.

We found that the total number of behavioural strategies was associated with greater decreases in energy intake. Similar findings were previously reported in a meta-regression analysis for diet and exercise interventions in individuals with overweight or obesity in the general population using the latest BCTTv1 [11], although this association was not seen in another report using the older version of BCT taxonomy [12]. This suggests that the BCTTv1 could be a useful tool in identifying active ingredients in lifestyle interventions to promote a reduction in energy intake. This is further supported by qualitative findings where postpartum women identified several strategies including self-monitoring of behaviour as helpful in changing diet and physical activity behaviours [51]. Future postpartum lifestyle interventions should focus on the delivery of effective behavioural strategies, particularly those identified for this group. There have not yet been postnatal interventions that were explicitly designed around these essential behavioural strategies in postpartum women.

Current and previous reviews found that the majority of behavioural strategies significantly associated with decreased energy intake are related to self-regulation, namely problem solving, goal setting of outcome, reviewing outcome goal, feedback on behaviour, and self-monitoring of behaviour [13]. Provision of information was not associated with energy intake in the current study. Previous systematic reviews in other populations [11,12,49] also identified that the behavioural strategies relating to self-regulation were positively associated with diet and physical activity behaviours. However, previous studies also identified provision of information as effective [11,12], which we did not find in postpartum women in the current study. To further explore the effect of self-regulation skills, we considered the presence of behavioural strategies as a group based on Control Theory. We found that behavioural strategies associated with Control Theory which facilitates self-regulation resulted in greater decreases in energy intake. These findings are consistent with recent evidence which suggests that lifestyle coaching approaches that support autonomous motivation are effective in producing behavioural change in the long-term [11,52]. This favouring of autonomous, self-regulatory strategies over information provision is also consistent with the known barriers to lifestyle modification in the postpartum period which are related to practical issues instead of knowledge deficit on lifestyle management [18]. Future interventions for postpartum women should focus on facilitating autonomy and self-regulation through approaches such as lifestyle coaching instead of didactic information provision.

We did not find any behavioural strategies to be significantly associated with physical activity. Previous meta-regression on behavioural strategies for physical activity did not analyse physical activity separately from dietary outcomes [11], and we were unable to compare our results. Dombrowski et al. (2012) conducted a meta-regression on physical activity in adults with obesity and obesity-related comorbidities and similarly failed to detect a significant association between behavioural strategies and physical activity [12]. Another study that investigated the associations between behavioural strategies and physical activity using paired Z-scores instead of meta-regression methods did report several strategies to be significantly associated with physical activity, including action planning, providing instruction, and reinforcing effort towards behaviour [14]. This has yet to be confirmed in a meta-regression. This knowledge gap highlights the need for more research on behavioural strategies to increase physical activity specifically in postpartum women considering their unique barriers and challenges such as lack of time and energy to be physically active at this life stage [18].

Despite several significant associations between behavioural strategies and energy intake reduction, we did not find any significant associations with weight loss. Subtle health behaviour changes such as reducing energy intake can occur without significant weight loss, as it takes a daily energy deficit of 2250 KJ to achieve a weight loss of 0.5 kg per week. Considering the overall modest energy reduction (600 kJ/day) seen in the current review, significant weight loss is an unlikely outcome. However, improved diet quality independent of weight changes could improve total and cause-specific mortality [53], and considering the high risk of weight gain in this population [54], a modest energy restriction to prevent further weight gain could support chronic diseases prevention [55]. Thus, behavioural strategies effective in reducing energy intake with or without significant weight loss could also be implemented in interventions for postpartum women with the aim of improving overall health.

### 4.3. Limitations

There are several limitations to this study. First, the absence of a behavioural strategy could reflect a reporting omission rather than an absence within the intervention delivered. Every effort was undertaken with this review to ensure that the study protocol was obtained and coded and that authors were contacted to collect missing information. Second, no included study reported their intervention according to BCTTv1 or another BCT taxonomy; therefore, all behavioural strategies were subjectively coded. To optimise reliability and accuracy, both coders completed the BCT taxonomy training and they were also dietitians experienced in developing lifestyle interventions for women. Third, significant heterogeneity remains unexplained in many subgroups, which suggests the contribution of other confounders. Fourth, there are likely to be significant interactions between the behavioural strategies. However, there was an insufficient number of studies in the current review to explore the interactions between clusters of behavioural strategies through a meta-classification and regression trees (CART) analysis [56]. Instead, we have considered a theoretical approach by considering the effects of clustering of behavioural strategies with Control Theory. Fifth, most of the included studies had an overall high risk of bias. However, this rating was mainly due to lack of blinding, which is not often feasible in lifestyle interventions where the intervention provider and participants are generally aware of treatment allocation due to the interactive nature of interventions. Sixth, although BCTTv1 has expanded considerably the scope of the taxonomy compared to its previous version, there may remain techniques that are not currently coded in BCTTv1 [57].

## 5. Conclusions

This systematic review and meta-analysis of lifestyle interventions in postpartum women found that the provision of certain strategies including problem-solving, goal-setting of outcome, reviewing outcome goal, feedback on behaviour, self-monitoring of behaviour, behavioural substitution, and credible source and that the inclusions of behavioural strategies consistent with Control Theory were associated with greater decreases in energy intake. Lifestyle intervention studies for postpartum women should consider including these strategies as part of the core components. Further research is required to identify effective behavioural strategies for increasing physical activity and for promoting weight loss in postpartum women.

## Figures and Tables

**Table 1:** Characteristics of included studies.

Study	Country	Sample Size	Intervention	Active Phase (week)	Risk Factor at Inclusion	Outcomes
Berry, 2015	USA	60	Diet and exercise	6 months	Baseline BMI ≥25 kg/m^2^	Physical activity
Bertz, 2015	Sweden	68	Diet and exercise	12 weeks	Prepregnancy 25–35 kg/m^2^	Weight, energy intake, physical activity
Colleran, 2012	USA	31	Diet and exercise	16 weeks	Baseline BMI 25–30 kg/m^2^	Weight, energy intake, physical activity
Craigie, 2011	UK	52	Diet and exercise	12 weeks	Baseline BMI ≥25 kg/m^2^	Weight, physical activity
Daley, 2015	UK	94	Exercise	6 months	Postnatal depression	Weight
Davenport, 2011	Canada	47	Diet and exercise	16 weeks	Baseline BMI ≥25 kg/m^2^ or retained ≥5 kg from pregnancy	Weight, energy intake
deRosset, 2013	USA	24	Diet and exercise	12 weeks	Prepregnancy BMI ≥25 kg/m^2^	Physical activity
Dritsa, 2009	Canada	88	Exercise	12 weeks	Postnatal depression	Physical activity
Fjeldsoe, 2010	Australia	88	Exercise	12 weeks	-	Physical activity
Holmes, 2018	Northern Ireland	60	Diet and exercise	6 months	Prepregnancy or baseline BMI ≥25 kg/m^2^, gestational diabetes	Weight
Huang, 2009	Taiwan	240	Diet and exercise	6 months	-	Weight
Huseinovic, 2016	Sweden	110	Diet	12 weeks	Baseline BMI ≥27 kg/m^2^	Weight, energy intake, physical activity
Keller, 2014	USA	139	Exercise	12 months	Baseline BMI 25–35 kg/m^2^	Weight, energy intake, physical activity
Kernot, 2019	Australia	120	Exercise	6 weeks	-	Physical activity
Khodabandeh, 2017	Iran	220	Diet and exercise	6 weeks	-	Physical activity
Krummel, 2010	USA	151	Diet and exercise	12 months	-	Weight, physical activity
Leermakers,1998	USA	90	Diet and exercise	6 months	Baseline BMI ≥22 kg/m^2^ and retained ≥6.8 kg from pregnancy	Weight
Lioret, 2012	Australia	542	Diet and exercise	15 months	-	Physical activity
Lovelady, 2000	USA	48	Diet and exercise	12 weeks	Baseline BMI 25–30 kg/m^2^	Weight, energy intake, physical activity
Lovelady, 1995	USA	38	Exercise	10 weeks	-	Weight, energy intake, physical activity
Lovelady, 2009	USA	24	Exercise	16 weeks	Baseline BMI 20–30 kg/m^2^	Weight, energy intake, physical activity
Maturi, 2011	Iran	70	Exercise	12 weeks	-	Weight, physical activity
McCrory,1999	USA	68	Diet and exercise	11 days	-	Weight, physical activity
McIntyre, 2012	Australia	28	Exercise	12 weeks	Gestational diabetes	Weight
Nicklas, 2014	USA	75	Diet and exercise	12 months	Gestational diabetes	Weight
Ostbye, 2009	USA	450	Diet and exercise	9 months	Prepregnancy BMI ≥25 kg/m^2^	Weight, energy intake, physical activity
O’Toole, 2003	USA	40	Diet and exercise	12 weeks	Prepregnancy BMI 25–29.9 kg/m^2^	Weight, energy intake, physical activity
Parsa, 2017	Iran	120	Diet and exercise	3 weeks	-	Physical activity
Tripette, 2014	Japan	34	Exercise	40 days	Baseline BMI >22 kg/m^2^	Weight, energy intake, physical activity
Wiltheiss, 2012	USA	400	Diet and exercise	8 months	Prepregnancy and baseline BMI ≥25 kg/m^2^	Weight, energy intake
Youngwanichsetha, 2013	Thailand	69	Exercise	12 weeks	Type 2 diabetes	Weight
Zilberman, 2018	Israel	180	Diet and exercise	24 months	Gestational diabetes	Weight, physical activity
Zourladani, 2015	Greece	42	Exercise	12 weeks	-	Weight, physical activity

**Table 2:** Univariate meta-regression for energy intake in lifestyle interventions for postpartum women by behavioural strategies (k = 12).

Behavioural Strategies	β	95% Confidence Interval	P-Value	Adjusted R-Squared (%)
Total number of behavioural strategies	−0.36	−0.65, −0.07	0.02	49.40
Behavioural strategies consistent with control theory	−0.56	−1.12, −0.20	0.01	62.15
1.2 Problem solving	−1.74	−3.43, −0.05	0.05	32.33
1.3 Goal setting (outcome)	−1.91	−3.78, −0.04	0.05	39.72
1.4 Action planning	−0.69	−3.14, 1.77	0.55	0.00
1.7 Reviewing outcome goal	−3.94	−6.51, −1.36	0.01	72.05
2.2 Feedback on behaviour	−2.81	−4.26, −1.37	0.00	87.59
2.3 Self-monitoring of behaviour	0.44	−1.51, 2.38	0.63	0.00
2.4 Self-monitoring of outcome of behaviour	−3.20	−5.06, −1.33	0.00	80.22
2.5 Monitoring of outcome of behaviour without feedback	−0.57	−3.23, 2.10	0.65	0.00
3.1 Social support (unspecified)	−1.21	−2.97, 0.55	0.16	9.49
3.2 Social support (practical)	−0.75	−2.81, 1.31	0.44	0.00
4.1 Instructions on how to perform the behaviour	0.45	−1.52, 2.48	0.62	0.00
5.1 Information about health consequences	−1.71	−5.44, 2.01	0.33	2.61
6.1 Demonstration of the behaviour	1.22	−0.80, 3.25	0.21	5.91
8.1 Behavioural practice/rehearsal	0.87	−1.01, 2.76	0.33	0.00
8.2 Behaviour substitution	−3.20	−5.06, −1.33	0.00	80.22
8.7 Graded tasks	0.84	−1.03, 2.71	0.34	0.00
9.1 Credible source	−1.73	−3.28, −0.17	0.03	38.81
11.2 Reduce negative emotions	0.31	−3.06, 3.68	0.84	0.00
12.5 Adding objects to the environment	0.40	−1.57, 2.36	0.66	0.00
13.1 Identification of self as a role model	0.31	−3.06, 3.68	0.84	0.00

## Supplementary Materials

The following are available online at https://www.mdpi.com/2077-0383/9/1/237/s1, Figure S1: Forest plots and funnel plots for weight, energy intake, and physical activity, Table S1: PRISMA statement, Table S2: Search strategies*, Table S3: Inclusion and exclusion criteria of the studies*, Table S4: Behavioural strategies consistent with Control Theory, Table S5: Characteristics of included studies; Table S6: Risk of bias in individual studies, Table S7: Univariate meta-regression for body weight in lifestyle interventions for postpartum women by behavioural strategies (k = 25), Table S8: Univariate meta-regression for physical activity in lifestyle interventions for postpartum women by behavioural strategies (k = 24).

## References

[1] Althuizen E., van Poppel M.N., de Vries J.H., Seidell J.C., van Mechelen W. (2011). Postpartum behaviour as predictor of weight change from before pregnancy to one year postpartum. BMC Public Health.

[2] Boghossian N.S., Yeung E.H., Lipsky L.M., Poon A.K., Albert P.S. (2013). Dietary patterns in association with postpartum weight retention. Am. J. Clin. Nutr..

[3] Endres L.K., Straub H., McKinney C., Plunkett B., Minkovitz C.S., Schetter C.D., Ramey S., Wang C., Hobel C., Raju T. (2015). Postpartum weight retention risk factors and relationship to obesity at 1 year. Obstet. Gynecol..

[4] Olson C.M., Strawderman M.S., Hinton P.S., Pearson T.A. (2003). Gestational weight gain and postpartum behaviors associated with weight change from early pregnancy to 1 y postpartum. Int. J. Obes..

[5] Villamor E., Cnattingius S. (2006). Interpregnancy weight change and risk of adverse pregnancy outcomes: A population-based study. Lancet.

[6] Rooney B.L., Schauberger C.W., Mathiason M.A. (2005). Impact of perinatal weight change on long-term obesity and obesity-related illnesses. Obstet. Gynecol..

[7] Linne Y., Dye L., Barkeling B., Rossner S. (2003). Weight development over time in parous women—The spawn study-15 years follow-up. Int. J. Obes..

[8] Linne Y., Barkeling B., Rossner S. (2002). Natural course of gestational diabetes mellitus: Long term follow up of women in the spawn study. BJOG.

[9] Damschroder L.J., Aron D.C., Keith R.E., Kirsh S.R., Alexander J.A., Lowery J.C. (2009). Fostering implementation of health services research findings into practice: A consolidated framework for advancing implementation science. Implement. Sci..

[10] Lim S., Liang X., Hill B., Teede H., Moran L.J., O’Reilly S. (2019). A systematic review and meta-analysis of intervention characteristics in postpartum weight management using the tidier framework: A summary of evidence to implementation. Obes. Rev..

[11] Samdal G.B., Eide G.E., Barth T., Williams G., Meland E. (2017). Effective behaviour change techniques for physical activity and healthy eating in overweight and obese adults; systematic review and meta-regression analyses. Int. J. Behav. Nutr. Phys. Act..

[12] Dombrowski S.U., Sneihotta F.F., Avenell A., Johnston M., MacLennan G., Araujo-Soares V. (2012). Identifying active ingredients in complex behavioural interventions for obese adults with obesity-related co-morbidities or additional risk factors for co-morbidities: A systematic review. Health Psychol. Rev..

[13] Michie S., Abraham C., Whittington C., McAteer J., Gupta S. (2009). Effective techniques in healthy eating and physical activity interventions: A meta-regression. Health Psychol..

[14] Williams S.L., French D.P. (2011). What are the most effective intervention techniques for changing physical activity self-efficacy and physical activity behavior-and are they the same?. Health Educ. Res..

[15] Golley R.K., Hendrie G.A., Slater A., Corsini N. (2011). Interventions that involve parents to improve children’s weight-related nutrition intake and activity patterns—What nutrition and activity targets and behaviour change techniques are associated with intervention effectiveness?. Obes. Rev..

[16] Lara J., Evans E.H., O’Brien N., Moynihan P.J., Meyer T.D., Adamson A.J., Errington L., Sniehotta F.F., White M., Mathers J.C. (2014). Association of behaviour change techniques with effectiveness of dietary interventions among adults of retirement age: A systematic review and meta-analysis of randomised controlled trials. BMC Med..

[17] Nicklas J.M., Zera C.A., Seely E.W., Abdul-Rahim Z.S., Rudloff N.D., Levkoff S.E. (2011). Identifying postpartum intervention approaches to prevent type 2 diabetes in women with a history of gestational diabetes. BMC Pregnancy Childbirth.

[18] Carter-Edwards L., Ostbye T., Bastian L.A., Yarnall K.S., Krause K.M., Simmons T.J. (2009). Barriers to adopting a healthy lifestyle: Insight from postpartum women. BMC Res. Notes.

[19] Dasgupta K., Da Costa D., Pillay S., De Civita M., Gougeon R., Leong A., Bacon S., Stotland S., Chetty V.T., Garfield N. (2013). Strategies to optimize participation in diabetes prevention programs following gestational diabetes: A focus group study. PLoS ONE.

[20] Moher D., Liberati A., Tetzlaff J., Altman D.G., Group P. (2009). Preferred reporting items for systematic reviews and meta-analyses: The prisma statement. J. Clin. Epidemiol..

[21] Pietrobelli A., Agosti M., MeNu G. (2017). Nutrition in the first 1000 days: Ten practices to minimize obesity emerging from published science. Int. J. Environ. Res. Public Health.

[22] Michie S., Richardson M., Johnston M., Abraham C., Francis J., Hardeman W., Eccles M.P., Cane J., Wood C.E. (2013). The behavior change technique taxonomy (v1) of 93 hierarchically clustered techniques: Building an international consensus for the reporting of behavior change interventions. Ann. Behav. Med..

[23] Sterne J.A.C., Savovic J., Page M.J., Elbers R.G., Blencowe N.S., Boutron I., Cates C.J., Cheng H.-Y., Corbett M.S., Eldridge S.M. (2019). RoB 2: a revised tool for assessing risk of bias in randomized trials. BMJ..

[24] Harrer M., Cuijpers P., Furukawa T.A., Ebert D.D. Doing Meta-Analysis in R: A Hands-on Guide; 2019. https://bookdown.org/MathiasHarrer/Doing_Meta_Analysis_in_R/.

[25] deRosset L., Berry D.C., Sanchez-Lugo L., Ritter K., Purdum C., Santolim V., Gilliland R., Pender L. (2013). Mama sana... Usted sana: Lessons learned from a postpartum weight loss intervention for hispanic women with infants six months or less. Hisp. Health Care Int..

[26] Lioret S., Campbell K.J., Crawford D., Spence A.C., Hesketh K., McNaughton S.A. (2012). A parent focused child obesity prevention intervention improves some mother obesity risk behaviors: The melbourne infant program. Int. J. Behave. Nutr. Phys. Act..

[27] Haire-Joshu D.L., Schwarz C.D., Peskoe S.B., Budd E.L., Brownson R.C., Joshu C.E. (2015). A group randomized controlled trial integrating obesity prevention and control for postpartum adolescents in a home visiting program. Int. J. Behav. Nutr. Phys. Act..

[28] McCrory M.A., Nommsen-Rivers L.A., Mole P.A., Lonnerdal B., Dewey K.G. (1999). Randomized trial of the short-term effects of dieting compared with dieting plus aerobic exercise on lactation performance. Am. J. Clin. Nutr..

[29] Lovelady C.A., Bopp M.J., Colleran H.L., Mackie H.K., Wideman L. (2009). Effect of exercise training on loss of bone mineral density during lactation. Med. Sci. Sports Exerc..

[30] Khodabandeh F., Mirghafourvand M., KamaliFard M., Mohammad-Alizadeh-Charandabi S., Jafarabadi M.A. (2017). Effect of educational package on lifestyle of primiparous mothers during postpartum period: A randomized controlled clinical trial. Health Educ. Res..

[31] Parsa P., Alafchi N., Soltani F., Roshanaei G. (2017). Effects of group counselling on health-promoting behaviours in mothers during postpartum period: A randomised controlled trial. J. Clin. Diagn. Res..

[32] Zourladani A., Zafrakas M., Chatzigiannis B., Papasozomenou P., Vavilis D., Matziari C. (2014). The effect of physical exercise on postpartum fitness, hormone and lipid levels: A randomized controlled trial in primiparous, lactating women. Arch. Gynecol. Obstet..

[33] Youngwanichsetha S., Phumdoung S., Ingkathawornwong T. (2013). The effects of tai chi qigong exercise on plasma glucose levels and health status of postpartum thai women with type 2 diabetes. Focus Altern. Complement. Ther..

[34] Nicklas J.M., Zera C.A., England L.J., Rosner B.A., Horton E., Levkoff S.E., Seely E.W. (2014). A web-based lifestyle intervention for women with recent gestational diabetes mellitus: A randomized controlled trial. Obstet. Gynecol..

[35] Leermakers E.A., Anglin K., Wing R.R. (1998). Reducing postpartum weight retention through a correspondence intervention. Int. J. Obes..

[36] Ostbye T., Krause K.M., Lovelady C.A., Morey M.C., Bastian L.A., Peterson B.L., Swamy G.K., Brouwer R.J.N., McBride C.M. (2009). Active mothers postpartum. A randomized controlled weight-loss intervention trial. Am. J. Prev. Med..

[37] Fjeldsoe B.S., Miller Y.D., Marshall A.L. (2010). Mobilemums: A randomized controlled trial of an sms-based physical activity intervention. Ann. Behav. Med..

[38] Krummel D., Semmens E., MacBride A.M., Fisher B. (2010). Lessons learned from the mothers’ overweight management study in 4 west virginia wic offices. J. Nutr. Educ. Behav..

[39] Tripette J., Haruka M., Yuko G., Ryoko K., Azusa S., Satoshi H., Aiko H., Motohiko M. (2014). Home-based active video games to promote weight loss during the postpartum period. Med. Sci. Sports Exerc..

[40] Daley A.J., Blamey R.V., Jolly K., Roalfe A.K., Turner K.M., Coleman S., McGuinness M., Jones I., Sharp D.J., MacArthur C. (2015). A pragmatic randomized controlled trial to evaluate the effectiveness of a facilitated exercise intervention as a treatment for postnatal depression: The pam-pers trial. Psychol. Med..

[41] Huseinovic E., Bertz F., Brekke H.K., Winkvist A. (2018). Two-year follow-up of a postpartum weight loss intervention: Results from a randomized controlled trial. Matern. Child Nutr..

[42] Huseinovic E., Bertz F., Leu Agelii M., Hellebo Johansson E., Winkvist A., Brekke H.K. (2016). Effectiveness of a weight loss intervention in postpartum women: Results from a randomized controlled trial in primary health care. Am. J. Clin. Nutr..

[43] Kernot J., Lewis L., Olds T., Maher C. (2019). Effectiveness of a facebook-delivered physical activity intervention for postpartum women: A randomized controlled trial. J. Phys. Act. Health.

[44] Keller C., Ainsworth B., Records K., Todd M., Belyea M., Vega-Lopez S., Permana P., Coonrod D., Nagle-Williams A. (2014). A comparison of a social support physical activity intervention in weight management among post-partum latinas. BMC Public Health.

[45] Lovelady C.A., Garner K.E., Moreno K.L., Williams J.P. (2000). The effect of weight loss in overweight, lactating women on the growth of their infants. N. Engl. J. Med..

[46] Lovelady C.A., Stephenson K.G., Kuppler K.M., Williams J.P. (2006). The effects of dieting on food and nutrient intake of lactating women. J. Am. Diet. Assoc..

[47] Lovelady C.A., Williams J.P., Garner K.E., Moreno K.L., Taylor M.L., Leklem J.E. (2001). Effect of energy restriction and exercise on vitamin b-6 status of women during lactation. Med. Sci. Sports Exerc..

[48] Zilberman-Kravits D., Meyerstein N., Abu-Rabia Y., Wiznitzer A., Harman-Boehm I. (2018). The impact of a cultural lifestyle intervention on metabolic parameters after gestational diabetes mellitus a randomized controlled trial. Matern. Child Health J..

[49] Presseau J., Ivers N.M., Newham J.J., Knittle K., Danko K.J., Grimshaw J.M. (2015). Using a behaviour change techniques taxonomy to identify active ingredients within trials of implementation interventions for diabetes care. Implement. Sci..

[50] Lim S., Dunbar J.A., Versace V.L., Janus E., Wildey C., Skinner T., O’Reilly S. (2017). Comparing a telephone- and a group-delivered diabetes prevention program: Characteristics of engaged and non-engaged postpartum mothers with a history of gestational diabetes. Diabetes Res. Clin. Pract..

[51] Smith D.M., Taylor W., Lavender T. (2016). Behaviour change techniques to change the postnatal eating and physical activity behaviours of women who are obese: A qualitative study. BJOG.

[52] Rutten G.M., Meis J.J., Hendriks M.R., Hamers F.J., Veenhof C., Kremers S.P. (2014). The contribution of lifestyle coaching of overweight patients in primary care to more autonomous motivation for physical activity and healthy dietary behaviour: Results of a longitudinal study. Int. J. Behav. Nutr. Phys. Act..

[53] Sotos-Prieto M., Bhupathiraju S.N., Mattei J., Fung T.T., Li Y., Pan A., Willett W.C., Rimm E.B., Hu F.B. (2017). Association of changes in diet quality with total and cause-specific mortality. N. Engl. J. Med..

[54] Adamson L., Brown W., Byles J., Chojenta C., Dobson A., Fitzgerald D., Hockey R., Loxton D., Powers J., Spallek M. Women’s Weight: Findings from the Australian Longitudinal Study on Women’s Health: Report Prepared for the Australian Government Department of Health and Ageing; 2007. https://www.alswh.org.au/images/content/pdf/major_reports/2007_major_report_b.pdf.

[55] Zheng Y., Manson J.E., Yuan C., Liang M.H., Grodstein F., Stampfer M.J., Willett W.C., Hu F.B. (2017). Associations of weight gain from early to middle adulthood with major health outcomes later in life. JAMA.

[56] Dusseldorp E., van Genugten L., van Buuren S., Verheijden M.W., van Empelen P. (2014). Combinations of techniques that effectively change health behavior: Evidence from meta-cart analysis. Health Psychol..

[57] Gillison F.B., Rouse P., Standage M., Sebire S.J., Ryan R.M. (2019). A meta-analysis of techniques to promote motivation for health behaviour change from a self-determination theory perspective. Health Psychol. Rev..

