# Supplementary material for: What Are the Most Effective Behavioural Strategies in Changing Postpartum Women’s Physical Activity and Healthy Eating Behaviours? A Systematic Review and Meta-Analysis
Source: J Clin Med. 2020 Jan 16;9(1):237. doi: 10.3390/jcm9010237 (PMC7019954; doi:10.3390/jcm9010237)

Supplementary file

What Are the Most Effective Behavioural Strategies in Changing Postpartum Women's Physical Activity and Healthy Eating Behaviours? A Systematic Review and Meta-Analysis

Siew Lim [1*], Briony Hill [1], Stephanie Pirotta [1], Sharleen O'Reilly [2#] and Lisa Moran [1#,]

[1] Monash Centre for Health Research and Implementation, Monash University, Clayton, Vic, Australia University; briony.hill@monash.edu; stephanie.pirotta@monash.edu; lisa.moran@monash.edu

[2] School of Agriculture and Food Science, University College Dublin, Belfield, Dublin, Ireland; harleen.oreilly@ucd.ie

* Correspondence: siew.lim1@monash.edu

\# These authors contributed equally

Table S1: Preferred Reporting Items for Systematic Reviews and Meta-Analyses (PRISMA) statement.

Section/topic	#	Checklist item	Reported on page #
**TITLE**			
Title	1	Identify the report as a systematic review, meta-analysis, or both.	1
**ABSTRACT**			
Structured summary	2	Provide a structured summary including, as applicable, background; objectives; data sources; study eligibility criteria, participants, and interventions; study appraisal and synthesis methods; results; limitations; conclusions and implications of key findings; and systematic review registration number.	2
**INTRODUCTION**			
Rationale	3	Describe the rationale for the review in the context of what is already known.	3-4
Objectives	4	Provide an explicit statement of questions being addressed with reference to participants, interventions, comparisons, outcomes, and study design (PICOS).	4
**METHODS**			
Protocol and registration	5	Indicate if a review protocol exists and if and where it can be accessed (e.g., Web address), and if available, provide registration information including registration number.	4
Eligibility criteria	6	Specify study characteristics (e.g., PICOS and length of followup), and report characteristics (e.g., years considered, language, and publication status) used as criteria for eligibility, giving rationale.	5; Additional Files
Information sources	7	Describe all information sources (e.g., databases with dates of coverage and contact with study authors to identify additional studies) in the search and date last searched.	4-5
Search	8	Present full electronic search strategy for at least one database, including any limits used, such that it could be repeated.	Additional Files
Study selection	9	State the process for selecting studies (i.e., screening, eligibility, included in systematic review, and, if applicable, included in the meta-analysis).	5
Data collection process	10	Describe method of data extraction from reports (e.g., piloted forms and independently, in duplicate) and any processes for obtaining and confirming data from investigators.	5-6
Data items	11	List and define all variables for which data were sought (e.g., PICOS and funding sources) and any assumptions and simplifications made.	5-6
Risk of bias in individual studies	12	Describe methods used for assessing risk of bias of individual studies (including specification of whether this was done at the study or outcome level) and how this information is to be used in any data synthesis.	6
Summary measures	13	State the principal summary measures (e.g., risk ratio and difference in means).	6
Synthesis of results	14	Describe the methods of handling data and combining results of studies, if done, including measures of consistency (e.g., I^2) for each meta-analysis.	6

| Section/topic | # | Checklist item | Reported on page # |

Risk of bias across studies	15	Specify any assessment of risk of bias that may affect the cumulative evidence (e.g., publication bias and selective reporting within studies).	6
Additional analyses	16	Describe methods of additional analyses (e.g., sensitivity or subgroup analyses and meta-regression), if done, indicating which were prespecified.	6
**RESULTS**			
Study selection	17	Give numbers of studies screened, assessed for eligibility, and included in the review, with reasons for exclusions at each stage, ideally with a flow diagram.	7; Figure 1
Study characteristics	18	For each study, present characteristics for which data were extracted (e.g., study size, PICOS, and followup period), and provide the citations.	7; Table 1
Risk of bias within studies	19	Present data on risk of bias of each study and, if available, any outcome level assessment (see item 12).	7-8; Figure 2, Additional Files
Results of individual studies	20	For all outcomes considered (benefits or harms), presen, for each study (a) simple summary data for each intervention group and (b) effect estimates and confidence intervals, ideally with a forest plot.	Additional Files
Synthesis of results	21	Present results of each meta-analysis done, including confidence intervals and measures of consistency.	8-9; Additional Files
Risk of bias across studies	22	Present results of any assessment of risk of bias across studies (see item 15).	7; Additional Files
Additional analysis	23	Give results of additional analyses, if done (e.g., sensitivity or subgroup analyses and meta-regression (see item 16)).	8-9, Additional Files
**DISCUSSION**			
Summary of evidence	24	Summarize the main findings including the strength of evidence for each main outcome; consider their relevance to key groups (e.g., healthcare providers, users, and policy makers).	9
Limitations	25	Discuss limitations at the study and outcome levels (e.g., risk of bias) and at the review level (e.g., incomplete retrieval of identified research, reporting bias).	11–12
Conclusions	26	Provide a general interpretation of the results in the context of other evidence and implications for future research.	12
**FUNDING**			
Funding	27	Describe sources of funding for the systematic review and other support (e.g., supply of data) and the role of funders for the systematic review.	1

Figure 2009. Preferred Reporting Items for Systematic Reviews and Meta-Analyses: The PRISMA Statement. PLoS Med 6(7): e1000097. doi:10.1371/journal.pmed1000097.

Table S2: Search strategies.

1. (MH "Postpartum Period+")
2. TI (postpartum OR post-partum OR postnatal OR post-natal OR puerperium OR postpartal OR post-partal OR lactating OR lactation OR "nursing women" OR breastfeeding OR breast-feeding OR "after birth" OR "following pregnancy" OR postpregnancy OR "post pregnancy" OR "following childbirth" OR "after delivery" OR "post childbirth") OR AB (postpartum OR post-partum OR postnatal OR post-natal OR puerperium OR postpartal OR post-partal OR lactating OR lactation OR "nursing women" OR breastfeeding OR breast-feeding OR "after birth" OR "following pregnancy" OR postpregnancy OR "post pregnancy" OR "following childbirth" OR "after delivery" OR "post childbirth")
3. 1 or 2
4. TI diet* OR AB diet*
5. TI (life*style N2 (chang* OR intervention*)) OR AB (life*style N2 (chang* OR intervention*))
6. TI ("physic* activ*" OR exercis*) OR AB ("physic* activ*" OR exercis*)
7. 4 or 5 or 6
8. 3 and 7
9. (MH "Randomized Controlled Trial+")
10. (MH "Clinical Trial+")
11. randomi?ed controlled trial$.tw.
12. RCT.tw.
13. random allocation.tw.
14. randomly allocated.tw.
15. allocated randomly.tw.
16. (allocated adj2 random).tw.
17. or/9–16
18. 8 and 17

Limit: Humans

*As a parallel body of work, these were included in another paper previously published at *Obesity Reviews*. "A systematic review and meta-analysis of intervention characteristics in postpartum weight management using the TIDieR framework: A summary of evidence to inform implementation. Lim S, Liang X, Hill B, Teede H, Moran LJ, O'Reilly S. Obesity Reviews. 2019, 20(7):1045-1056".

Table S3. Inclusion and exclusion criteria of the systematic review and meta-analysis of lifestyle intervention in postpartum women.

	Participants (P)	Intervention (I)/ Exposure	Comparison (C)	Outcomes (O)	Study type	Limits
**Inclusion criteria**	Postpartum women (2 years post delivery).	Dietary, physical activity, or behavioral interventions<br><br>Theoretical framework, type, duration, sessions/contact, location, technology, self-monitoring, intervention provider, behavioural strategies, and group/individual<br><br>Sampling frame, age, baseline BMI inclusion and exclusion criteria, medical history, diet history, physical activity status, breastfeeding status, and withdrawals/losses to followup	Usual care, no intervention, or minimal intervention (single session at baseline)	Weight or weight change BMI or BMI change; total energy intake or change; physical activity or change; attrition; and compliance	RCT	All languages; translation will be obtained whenever possible; and not limited by year
**Exclusion criteria**	Pregnant women	Allergen avoidance studies, acute studies, and supplement trials; intervention that recruited during pregnancy<br><br>Exercise intervention focusing only on pelvic floor exercise and urinary incontinence<br><br>Intervention focusing only on initiating or increasing breastfeeding (without diet or exercise component)	Any dietary or physical activity intervention in the control arm that provides more contact than a single baseline information session	Studies without relevant outcomes	Editorial, narrative review, conference abstract, letters, commentaries, uncontrolled trials, study protocol, and non-randomized controlled trials; studies with pregnant women will only be included if subgroup data is available for postpartum women	

Table S4: Behavioural strategies consistent with Control Theory.

1. Goals and planning

1.1. Goal setting (behaviour)
1.2. Problem solving
1.3. Goal setting (outcome)
1.4. Action planning
1.5. Reviewing behaviour goal(s)
1.6. Discrepancy between current behaviour and goal
1.7. Reviewing outcome goal(s)
1.8. Behavioral contract
1.9. Commitment

2. Feedback and monitoring

2.1. Monitoring of behaviour by others without feedback
2.2. Feedback on behaviour
2.3. Self-monitoring of behaviour
2.4. Self-monitoring of outcome(s) of behaviour
2.5. Monitoring of outcome(s) of behaviour without feedback
2.6. Biofeedback
2.7. Feedback on outcome(s) of behaviour

Table S5: Characteristics of included studies

Study; sample size	Country	Ethnicity	Postpartum age; postpartum population	Duration; number of sessions
Berry 2015<br>N = 60	USA	77% African-American; 23% Non-Hispanic White	At least 6 weeks postpartum;<br>BMI > 25 kg/m^2	6 months<br>In-person: 15
Bertz 2015<br>N = 68	Sweden	n/a	10–14 weeks postpartum;<br>Prepregnancy BMI 25–35 kg/m^2	12 weeks<br>In-person: 4; SMS: 24
Colleran 2012<br>N = 31	USA	85% White, Non-Hispanic; 11% African-American;<br>4% Hispanic	4 weeks postpartum;<br>BMI 25 to 30 kg/m^2	16 weeks<br>Session number could not be determined
Craigie 2011<br>N = 52	UK	93–96% Caucasian	6–18 months postpartum;<br>BMI > 25 kg/m^2	12 weeks<br>In-person: 3; Phone: 3
Daley 2015<br>N = 94	UK	57–68% white	Within 6 months postpartum;<br>depression according to ICD-10 and EPDS	6 months<br>In-person: 2; Phone: 2

Davenport 2011<br>N = 47	Canada	85–90% Caucasian	7–9 weeks postpartum;<br>BMI ≥ 25.0 kg/m^2 and/or had retained ≥5.0 kg from pregnancy	16 weeks<br>48–64 walking sessions
deRosset 2013<br>N = 24	USA	100% Hispanic	6 weeks postpartum;<br>overweight or obese by self-report according to prepregnancy BMI	12 weeks<br>In-person: 12
Dritsa 2009<br>N = 88	Canada	n/a	4–38 weeks postpartum;<br>EPDS ≥ 10	12 weeks<br>In-person: 4
Fjeldsoe 2010<br>N = 88	Australia	2–6% identified as an Aboriginal or Torres Strait Islander	Less than 12 months postpartum;<br>general population	12 weeks<br>In-person: 2; SMS: 47–71
Holmes et al. 2018	USA	Caucasian	24 weeks postpartum;<br>postnatal overweight with PH of GDM	3 months
Huang 2009<br>N = 240	Taiwan	n/a	24–48 hours to 6 months postpartum;<br>general population	6 months<br>In-person: 3
Huseinovic 2016<br>N = 110	Sweden	n/a	6–15 weeks postpartum;<br>BMI ≥ 27 kg/m^2	12 weeks<br>In-person: 1; Text message: 12; Phone: 12
Keller 2014<br>N = 139	USA	100% Latina	6 weeks to less than 6 months postpartum;<br>BMI ≥ 25 kg/m^2	12 months<br>In-person: 52
Kernot et al. 2019	Aust	n/a	6 week to 6 month postpartum;<br>postpartum (facebook)	6 weeks<br>Weekly emails
Khodabandeh 2017<br>N = 220	Iran	99–100% Azeri	Day of discharge postpartum;<br>general population	6 weeks<br>In-person: 2; Text message: ~ 8
Krummel 2010<br>N = 151	USA	90% Caucasian	Up to 2 years postpartum;<br>general population	12 months<br>In-person: 11
Leermakers 1998<br>N = 90	USA	95–98% Caucasian	3–12 months postpartum;<br>exceeded their prepregnancy weight by at least 6.8 kg	6 months<br>In-person: 2; Phone: 12–24
Lioret 2012<br>N = 542	Australia	Country of Birth, 79% Australia; 21% Other	18 months postpartum;<br>general population	15 months<br>In-person: 6
Lovelady 2000<br>N = 48	USA	80–84% White; 16–19% Black	4 weeks postpartum;<br>BMI 25 to 30 kg/m^2	10 weeks<br>In-person: 40
Lovelady 1995<br>N = 38	USA	n/a	6 weeks postpartum;<br>general population	12 weeks<br>In-person: 60
Lovelady 2009	USA	95% Non-Hispanic White; 5% Asian	3 weeks postpartum;	16 weeks

N = 24			BMI 20 to 30 kg/m^2	In-person: 48
Maturi 2011<br>N = 70	Iran	n/a	6 weeks to 6 months postpartum;<br>BMI > 19.8 and < 29 kg/m^2	12 weeks<br>In-person: 1; Text message: 12; Phone: 6
McCrory 1999<br>N = 68	USA	77–82% Non-Hispanic White; 9–14% Hispanic; 0–13% Black; 0–9% Asian	12 ± 4 weeks postpartum;<br>general population	11 days
McIntyre 2012<br>N = 28	Australia	n/a	6 weeks postpartum;<br>post-gestational diabetes	12 weeks<br>In-person: 1; Phone: 8
Nicklas 2014<br>N = 75	USA	51–64% White; 25–36% African American; 11–13% Asian; 15–25% Hispanic or Latina	6 weeks postpartum;<br>post-gestational diabetes	12 months;<br>cannot be determined
Ostbye 2009<br>N = 450	USA	52-53% White; 45% Black; 2–3% Asian/Other	6 weeks postpartum;<br>prepregnancy BMI ≥ 25 kg/m^2	9 months<br>In-person: 18<br>Phone: 6
O'Toole 2003<br>N = 40	USA	98% Caucasian; 3% African American	6 weeks to 6 months postpartum;<br>prepregnancy BMI 25–29.9 kg/m^2	Cannot be determined
Parsa 2017<br>N = 120	Iran	n/a	3–20 days postpartum;<br>general population	3 weeks<br>In-person: 3
Tripette 2014<br>N = 34	Japan	100% Japanese	3 months to 1 year postpartum;<br>BMI > 22 kg/m^2	40 days<br>In-person: 2
Wiltheiss 2012<br>N = 400	USA	75% white; 22% black; 4% other races; 5% Hispanic	Within 6 months postpartum;<br>BMI ≥ 25 kg/m^2	8 months<br>In-person: 1; Mail: 8; Phone: 8
Youngwanichsetha 2013<br>N = 69	Thailand	n/a	6–12 weeks postpartum;<br>type 2 diabetes	12 weeks<br>In-person: 3
Zourladani 2015<br>N = 42	Greece	100% Greek	4–6 weeks postpartum;<br>general population	12 weeks<br>In-person: 36
Zilberman et al. 2018	Israel	Jewish and Bedouin	3–4 months postpartum;<br>general population	24 months<br>3 individual sessions<br>4 groups

Table S6: Risk of bias of included studies*.

Author	Randomisation process	Deviations from intended interventions	Missing outcome data	Measurement of the outcome	Selection of the reported result	Overall bias
Berry 2015	Low	High	Low	High	Low	High
Bertz 2015	Low	High	Low	Low	Low	High
Colleran 2012 MyPyramid	Low	High	Low	High	Low	High
Craigie 2011	Low	High	Low	Low	Low	High
Davenport 2011	Some concerns	High	Low	High	Low	High
deRosset 2013	Low	High	Some concerns	High	Low	High
Dritsa 2009	Some concerns	High	Some concerns	High	Low	High
Fjeldsoe 2010	Low	High	Low	High	Low	High
Holmes 2018	Low	High	High	High	Low	High
Huang 2011	Some concerns	High	Low	High	Low	High
Huseinovic 2016	Low	High	Low	High	Low	High
Keller 2014	Some concerns	High	High	Low	Low	High
Kernot 2019	Low	High	Some concerns	High	High	High
Khodabandeh 2017	Low	High	Low	High	Low	High
Krummel 2010	Some concerns	High	Low	High	Low	High
Leermakers 1998	Some concerns	High	Low	High	Low	High
Lioret 2012	Low	High	Low	High	Low	High
Lovelady 2000	Some concerns	High	High	Low	Low	High
Lovelady 1995	Some concerns	High	Low	Low	Low	High
Lovelady 2009	Some concerns	High	Low	High	Low	High
Maturi 2011	Low	High	Low	High	Low	High
McCrory 1999	Low	High	Low	High	Low	High
McIntyre 2012	Some concerns	High	Low	High	Low	High
Nicklas 2014	Low	Low	Low	Low	Low	Low
Ostbye 2009	Some concerns	High	Low	High	Low	High
O'Toole 2003	Low	High	Low	High	Low	High
Parsa 2017	Low	High	Low	High	Low	High
Tripette 2014	Some concerns	Some concerns	Low	High	Low	High
Wiltheiss 2013	Low	High	Some concerns	High	Low	High
Youngwanichsetha 2013	Low	High	Low	Low	Low	High
Zilberman 2018	Some concerns	High	Some concerns	Some concerns	High	High
Zourdalani 2015	Low	High	Low	Low	Low	High

[1]Based on the Revised Cochrane risk of bias tool for randomized trials (RoB 2.0)

[2]Low: The study is judged to be at low risk of bias for all domains; Some concerns: The study is judged to be at some concern in at least one domain for this result; High: The study is judged to be at high risk of bias in at least one domain

for this result, or the study is judged to have some concerns for multiple domains in a way that substantially lowers confidence in the result.

Table S7. Univariate meta-regression for body weight in lifestyle interventions for postpartum women by behavioural strategies (k = 25).

**Behavioural strategies**	**β**	**95% Confidence interval**	**P-value**	**Adjusted R-squared (%)**
Total number of behavioural strategies	−0.19	−0.68, 0.31	0.45	0
Behavioural strategies consistent with control theory	−0.40	−1.12, 0.33	0.27	1.98
1.2 Problem solving	0.48	−1.97, 2.92	0.69	0
1.3 Goal setting (outcome)	−1.88	−5.06, 1.31	0.24	4.05
1.4 Action planning	0.31	−2.17, 2.78	0.80	0
1.7 Reviewing outcome goal	0.17	−5.98, 6.32	0.95	0
2.2 Feedback on behaviour	−1.79	−5.66, 2.08	0.35	0
2.3 Self-monitoring of behaviour	−1.99	−4.26, 0.29	0.63	14.99
2.4 Self-monitoring of outcome of behaviour	−1.06	−4.06, 1.93	0.47	0
2.5 Monitoring of outcome of behaviour without feedback	−2.34	−6.57, 1.89	0.26	2.80
2.7 Feedback on outcome(s) of behavior	0.55	−5.58, 6.67	0.85	0
3.1 Social support (unspecified)	1.69	−0.65, 4.03	0.15	5.30
3.2 Social support (practical)	−1.68	−4.52, 1.15	0.23	4.42
4.1 Instructions on how to perform the behaviour	0.48	−1.99, 2.95	0.69	0
5.1 Information about health consequences	−2.87	−8.35, 2.60	0.29	1.35
5.3 Information about social and environmental consequences	0.61	−4.00, 5.22	0.79	0
6.1 Demonstration of the behaviour	−1.77	−4.91, 1.38	0.26	0.87
7.1 Prompts/cues	2.57	−3.34, 8.48	0.38	0
8.1 Behavioural practice/rehearsal	0.57	−2.15, 3.28	0.67	0
8.2 Behaviour substitution	−1.64	−6.38, 3.09	0.48	0
8.7 Graded tasks	−1.34	−3.71, 1.03	0.26	4.28
9.1 Credible source	−0.16	−2.65, 2.33	0.90	0
9.2 Pros and cons	2.57	−3.34, 8.48	0.38	0
11.2 Reducing negative emotions	0.98	−2.59, 4.54	0.58	0
12.5 Adding objects to the environment	0.57	−2.06, 3.19	0.66	0
13.1 Identifying self as a role model	1.76	−3.66, 7.17	0.51	0

β = regression coefficient, CI = confidence interval; k = number of evaluations; adjusted R^2 = adjusted proportion of heterogeneity accounted for by moderator

Table S8. Univariate meta-regression for physical activity in lifestyle interventions for postpartum women by behavioural strategies (k = 24).

Behavioural strategies	β	95% Confidence interval	P-value	Adjusted R-squared (%)
Total number of behavioural strategies	−0.10	−0.21, 0.02	0.09	11.88
Behavioural strategies consistent with control theory	−0.14	−0.34, 0.06	0.16	6.70
1.1 Goal setting (behaviour)	-0.0560	−1.28, 1.17	0.93	0
1.2 Problem solving	−0.06	−1.28, 1.17	0.93	0
1.3 Goal setting (outcome)	−0.51	−1.34, 0.32	0.22	3.13
1.4 Action planning	−0.47	−1.98, 1.03	0.52	0
1.5 Reviewing behavior goal(s)	−0.26	−1.12, 0.59	0.53	0
1.7 Reviewing outcome goal	−0.56	−2.58, 1.46	0.57	0
2.2 Feedback on behaviour	−0.38	−2.42, 1.65	0.70	0
2.3 Self-monitoring of behaviour	−0.56	−1.55, 0.43	0.25	2.17
2.4 Self-monitoring of outcome of behaviour	−0.44	−1.27, 0.38	0.28	0
2.5 Monitoring of outcome of behaviour without feedback	−0.54	−1.63, 0.56	0.32	0
3.1 Social support (unspecified)	−0.54	−2.69, 1.62	0.61	0
3.2 Social support (practical)	−0.78	−1.62, 0.05	0.07	12.96
4.1 Instructions on how to perform the behaviour	−0.33	−1.36, 0.71	0.52	0
5.1 Information about health consequences	−0.33	−1.18, 0.52	0.43	0
5.3 Information about social and environmental consequences	−0.12	−1.66, 1.42	0.87	0
5.6 Information about emotional consequences	−0.21	−1.32, 0.90	0.70	0
6.1 Demonstration of the behaviour	0.11	−2.00, 2.22	0.91	0
7.1 Prompts/cues	−0.15	−1.20, 0.90	0.77	0
8.1 Behavioural practice/rehearsal	−0.56	−1.78, 0.66	0.35	0
8.2 Behaviour substitution	0.30	−0.67, 1.27	0.53	0
8.7 Graded tasks	−0.06	−1.10, 0.97	0.90	0
9.1 Credible source	0.38	−0.48, 1.25	0.37	0
9.2 Pros and cons	−0.46	−1.32, 0.40	0.28	0.17
10.9 Self-reward	−0.17	−1.67, 1.33	0.82	0
11.2 Reducing negative emotions	−0.56	−2.58, 1.46	0.57	0
12.5 Adding objects to the environment	−0.51	−1.99, 0.97	0.48	0
13.1 Identifying self as a role model	−0.50	−1.59, 0.58	0.35	0
13.2 Framing/reframing	−0.69	−2.65, 1.28	0.48	0
15.4 Self-talk	−0.21	−1.71, 1.29	0.78	0

β = regression coefficient, CI = confidence interval; k = number of evaluations; adjusted R^2 = adjusted proportion of heterogeneity accounted for by moderator

Figure S1: Forest plots and funnel plots for weight, energy intake, and physical activity.

Forest plot for body weight

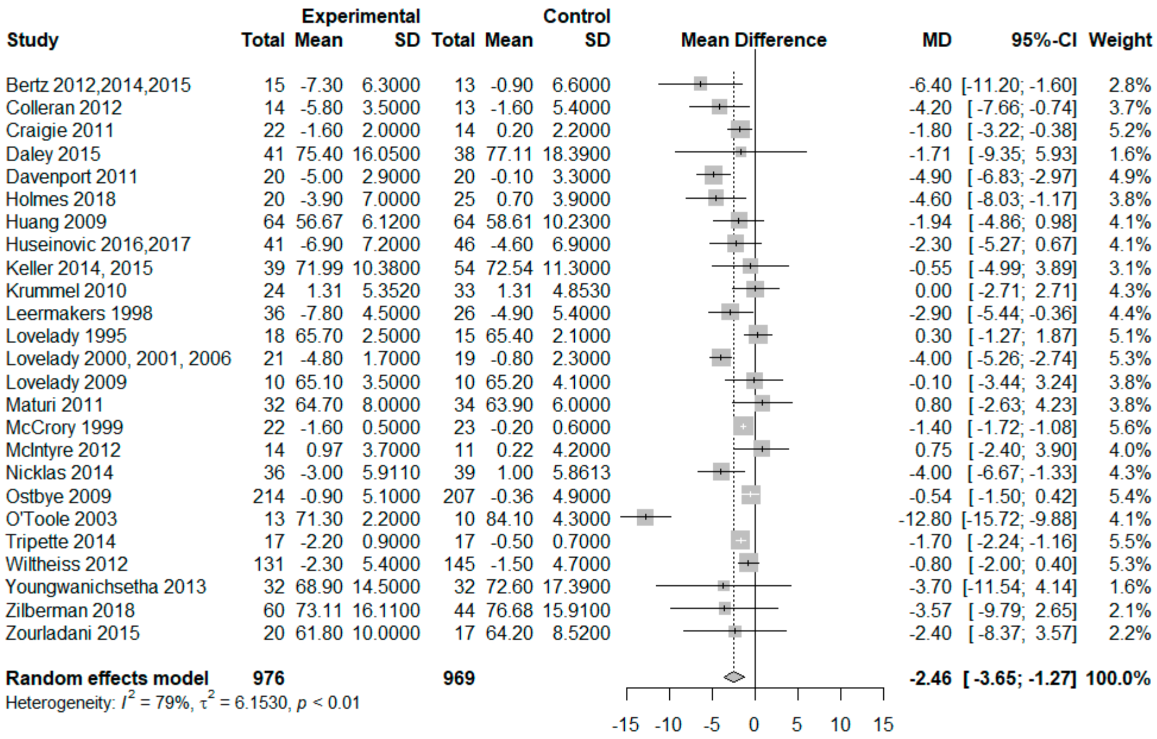

Forest plot for energy intake

Study	Experimental Total	Experimental Mean	Experimental SD	Control Total	Control Mean	Control SD	MD	95%-CI	Weight
Bertz 2012,2014,2015	15	-3.59	2.7820	13	-1.39	1.8660	-2.20	[-3.94; -0.46]	6.8%
Colleran 2012	14	-2.56	2.1799	13	-0.72	1.8200	-1.85	[-3.36; -0.34]	7.3%
Davenport 2011	20	7.90	1.5970	20	8.32	1.8640	-0.42	[-1.49; 0.66]	8.4%
Huseinovic 2016,2017	44	-2.75	3.2426	45	1.42	3.6777	-4.17	[-5.61; -2.73]	7.5%
Keller 2014, 2015	39	5.83	2.1548	54	5.42	1.6276	0.41	[-0.39; 1.22]	9.1%
Lovelady 1995	18	10.45	1.8243	15	9.22	1.3600	1.23	[0.14; 2.32]	8.4%
Lovelady 2000, 2001, 2006	19	-2.28	1.9707	16	-0.99	2.1255	-1.29	[-2.66; 0.08]	7.7%
Lovelady 2009	10	8.05	1.6539	10	7.07	1.5745	0.97	[-0.44; 2.39]	7.6%
Ostbye 2009	214	-0.87	2.2761	207	-0.70	2.2510	-0.16	[-0.60; 0.27]	9.7%
O'Toole 2003	13	6.66	0.3054	10	6.45	0.3724	0.21	[-0.07; 0.50]	9.9%
Tripette 2014	17	-0.86	2.3389	17	-0.11	1.0962	-0.75	[-1.98; 0.48]	8.1%
Wiltheiss 2012	131	7.36	2.1589	145	7.70	2.1757	-0.33	[-0.85; 0.18]	9.6%
**Random effects model**	**554**			**565**			**-0.61**	**[-1.53; 0.32]**	**100.0%**

Heterogeneity: $I^2 = 82\%$, $\tau^2 = 1.6501$, $p < 0.01$

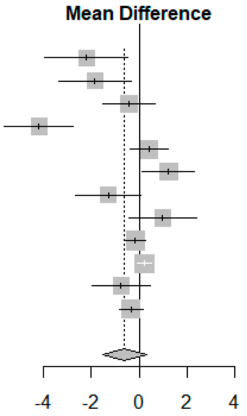

Forest plot for physical activity

Study	Experimental Total	Experimental Mean	Experimental SD	Control Total	Control Mean	Control SD	SMD	95%-CI	Weight
Berry	24	2.30	0.5000	20	1.90	0.6000	0.72	[0.10; 1.33]	4.2%
Bertz 2012,2014,2015	15	1588.00	2652.0000	13	766.00	3247.0000	0.27	[-0.48; 1.02]	3.9%
Colleran 2012	14	34.50	7.2000	13	33.80	7.2000	0.09	[-0.66; 0.85]	3.9%
Craigie 2011	22	19.00	47.0000	14	16.00	48.0000	0.06	[-0.61; 0.73]	4.1%
DeRosset	13	0.90	0.4900	11	0.70	0.5900	0.36	[-0.45; 1.17]	3.8%
Dritsa	46	124.09	96.3300	42	54.60	55.8000	0.87	[0.43; 1.30]	4.5%
Fjeldsoe	45	18.26	24.9400	43	16.36	25.5300	0.07	[-0.34; 0.49]	4.5%
Huseinovic 2016,2017	44	1053.00	2440.0000	45	394.00	2857.0000	0.25	[-0.17; 0.66]	4.5%
Keller 2014, 2015	39	6963.91	3126.5400	54	6425.04	3390.9000	0.16	[-0.25; 0.58]	4.5%
Kernot 2019	41	173.00	101.3000	40	160.00	77.4000	0.14	[-0.29; 0.58]	4.5%
Khodabandeh	105	16.00	15.2000	101	8.00	7.9000	0.65	[0.37; 0.93]	4.7%
Krummel 2010	18	705.00	2475.0000	24	308.00	2977.0000	0.14	[-0.47; 0.75]	4.2%
Lioret	178	387.72	346.2800	179	403.62	363.7900	-0.04	[-0.25; 0.16]	4.7%
Lovelady 1995	18	33.80	1.0000	15	28.90	1.1000	4.57	[3.21; 5.93]	2.8%
Lovelady 2000, 2001, 2006	21	4.50	4.9000	19	0.60	3.8000	0.87	[0.21; 1.52]	4.1%
Lovelady 2009	10	34.70	4.7434	10	34.40	4.1110	0.06	[-0.81; 0.94]	3.7%
Maturi 2011	32	11.00	34.4000	34	8.00	23.5000	0.10	[-0.38; 0.58]	4.4%
McCrory 1999	22	499.00	87.0000	23	135.00	126.0000	3.29	[2.37; 4.21]	3.6%
Ostbye 2009	214	21.80	143.6000	207	31.10	146.6000	-0.06	[-0.26; 0.13]	4.7%
O'Toole 2003	13	1981.00	72.0000	10	1902.00	79.0000	1.01	[0.13; 1.90]	3.7%
Parsa	56	17.91	3.0000	56	13.88	2.7100	1.40	[0.99; 1.81]	4.5%
Tripette 2014	17	25.90	3.3000	17	26.40	2.8000	-0.16	[-0.83; 0.51]	4.1%
Zilberman 2018	60	28.00	46.7000	44	1.00	2.3000	0.75	[0.35; 1.16]	4.5%
Zourladani 2015	20	1.96	0.3500	17	1.70	0.2600	0.82	[0.14; 1.49]	4.1%
**Random effects model**	**1087**			**1051**			**0.61**	**[0.20; 1.02]**	**100.0%**

Heterogeneity: $I^2 = 86\%$, $\tau^2 = 0.6588$, $p < 0.01$

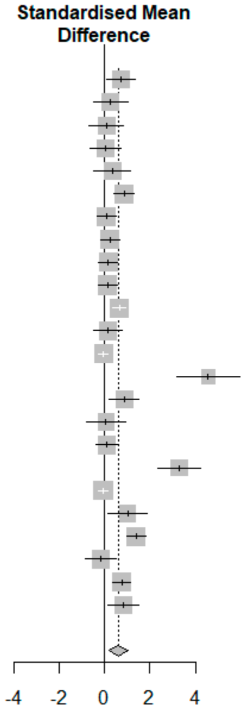

Funnel plot for body weight

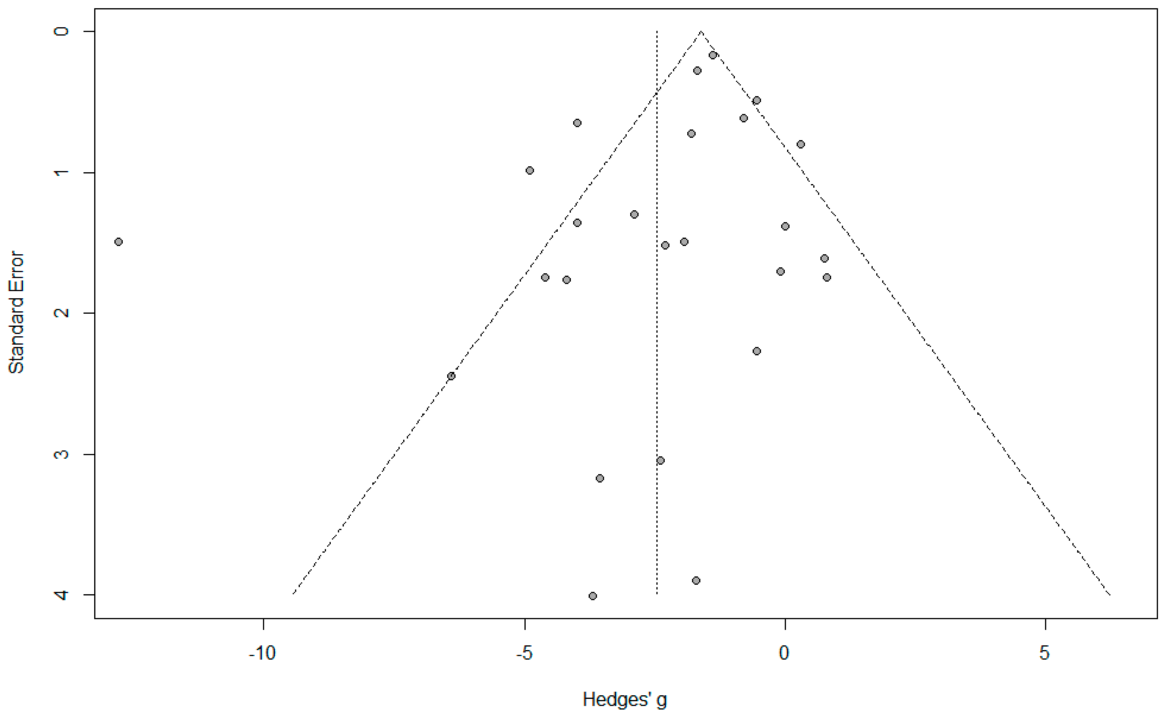

Funnel plot for energy intake

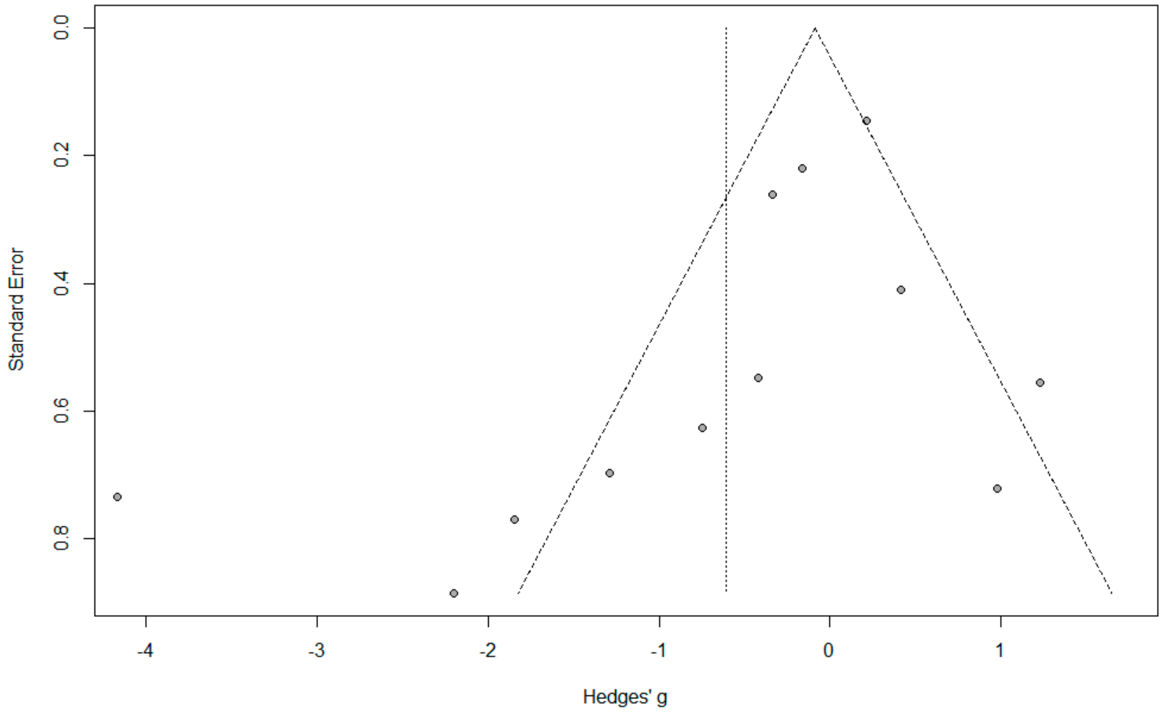

Funnel plot for physical activity

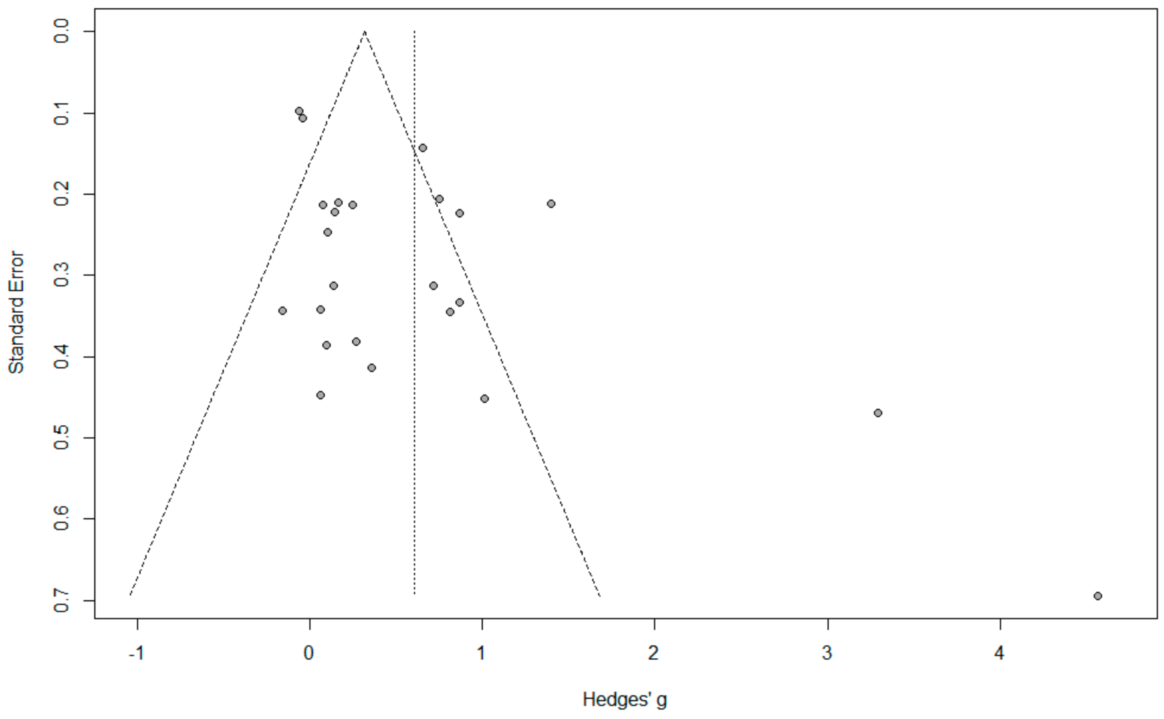

Supplement: Supplementary file 1 [file jcm-09-00237-s001.pdf]
